# Characteristics of deaths due to drug‐related causes among individuals recently released from prison in the United Kingdom, 1997–2025

**DOI:** 10.1111/add.70481

**Published:** 2026-05-14

**Authors:** Emmert Roberts, John Strang, Caroline Copeland

**Affiliations:** ^1^ National Addiction Centre King's College London, United Kingdom and the South London and the Maudsley NHS Foundation Trust London UK; ^2^ Faculty of Life Sciences and Medicine King's College London London UK

**Keywords:** accidental poisoning, drug‐related deaths, epidemiology, opioids, overdose, prison release

## Abstract

**Background and aims:**

There has been an extensive literature describing the elevated risk of drug‐related death following an individual's release from prison; however, few previous studies have reported nationally representative samples or provided granular detail as to which individual drugs were deemed implicated in death. We aimed to determine the (1) case characteristics, (2) circumstances of death and (3) type of implicated drugs among individuals recently released from prison and dying due to drug‐related causes, stratified by the length of time an individual had been released prior to death. We additionally aimed to determine any changes in the number of deaths and the percentage of deaths in which individual drugs were implicated over time.

**Design:**

Retrospective cohort study.

**Setting:**

Coronial records submitted voluntarily to the observational cohort National Programme on Substance Use Mortality (NPSUM) in the United Kingdom (UK), 1997–2025.

**Cases:**

Decedents who were recently released from prison up to one month prior to their death due to a drug‐related cause.

**Measurements:**

Information was available on decedent sociodemographics, characteristics of death and drugs implicated in death.

**Findings:**

A total of 597 decedents were found to have died due to a drug‐related cause following recent release from prison. Where records reported the exact timeframe between prison release and death (*n* = 309), 140 were reported to have died within two days of release, 81 between two days and one week and 88 between one week and one month. There was a statistically significant average annual percentage decrease of −3.9% [95% confidence interval (CI) = −6.6% to −1.0%, *P* = 0.006] of deaths following recent prison release within the NPSUM population over the studied timeframe. Overall, decedents were predominantly male (*n* = 547, 91.6%), of White ethnicity (*n* = 410, 68.7%) and had a mean age of 34.2 years (standard deviation 8.8; range 16–71). Accidental poisoning was the most common direct cause of death (*n* = 433, 72.5%), with opioids the most common class of implicated drug, involved in more than 90% of fatalities (*n* = 545, 91.3%).

**Conclusions:**

Over the past three decades relative to the overall UK National Programme on Substance Use Mortality cohort of deaths due to drug‐related causes, there have been substantial and reducing numbers of drug‐related fatalities among individuals recently released from prison. Accidental poisoning was the direct cause of death in almost three quarters of cases from 1997 to 2025, and opioids were consistently implicated in over 90% of fatalities.

## INTRODUCTION

There has been an extensive literature describing the substantial elevated risk of drug‐related death following an individuals' release from prison after a period of incarceration, a phenomenon often thought to be driven largely by a reduction in opioid tolerance during the period of imprisonment [1, 2, 3].

Although multiple previous observational studies have characterised this period of elevated risk of drug‐related death, few have reported nationally representative samples or have systematically examined any changes over extended time periods. Many previous studies also lack granular detail as to the specific circumstances of death, which individual drugs were ultimately deemed implicated or if distinct drugs or drug groups are more likely to be implicated at different time points following release [1, 4]. If specific case characteristics and/or particular drug use profiles were found to be associated with fatalities, this could lead to the development of updated guidance on priority interventions for this population, provide advocacy to link individuals with appropriate community treatment services post‐release and aid in the development of targeted harm reduction advice [5].

To our knowledge, no previous national epidemiological studies have examined fatalities because of drug‐related causes following recent release from prison in the United Kingdom (UK). To address this gap, we aimed to determine: (1) the case characteristics; (2) the circumstances of death; and (3) the number and type of drugs implicated in deaths among people dying because of drug‐related causes following release from prison, stratified by the length of time an individual had been released from prison prior to death. We additionally aimed to determine any changes in the number of deaths and the percentage of deaths in which individual drugs were implicated over time.

## METHODS

This study is reported according to the Strengthening the Reporting of Observational Studies in Epidemiology (STROBE) statement [6], the completed checklist available as Table S1 in the online Supporting information.

### The National Programme on Substance Use Mortality

We conducted a retrospective cohort study using records drawn from the National Programme on Substance Use Mortality (NPSUM), this paper containing recycled methods text material from previous NPSUM publications reported in accordance with guidance from the text recycling research project (https://textrecycling.org) [7]. This database contains information on an observational cohort that collects coronial data on deaths at any age and related to any psychoactive drug, other than nicotine, caffeine or where the sole substance implicated was alcohol [7]. In total, NPSUM holds records on more than 58 000 deaths with reports received from England, Wales, the Channel Islands and the Isle of Man since 1997. Additional reports were received from some coronial jurisdictions from the Scottish Crime and Drug Enforcement Agency between 2004 and 2011 and from the General Register Office for Northern Ireland since 2004, these deaths are included within the NPSUM cohort in their respective years. Coronial files typically include the coroner's decision as to cause of death, statements from witnesses, family and friends, first responders (e.g. police), general practitioner (GP) and hospital records that may include documentation of formal diagnoses and drug use history. Toxicology is discretionally requested by individual coroners, and coronial jurisdictions choose to voluntarily report all deaths within their area to NPSUM if any of the following criteria are met: (1) psychoactive substance(s) are directly implicated in death; (2) an individual has a history of dependence or problematic use of drugs; or (3) if controlled drugs are identified at post‐mortem. The King's College London (KCL) Biomedical and Health Sciences, Dentistry, Medicine and Natural and Mathematical Sciences Research Ethics Subcommittee (BDM RESC) re‐confirmed in August 2025 that NPSUM does not require Research Ethics Committee (REC) review as all individuals are deceased.

### Case identification

All cases reported between 1997 and August 2025 were identified and NPSUM fields searched for the following terms: *prison*, cell*, remand*, jail*, gaol*, custod*, police, inmate*, detention*, detain*, probation* and ‘HMP’ (i.e. His Majesty's Prison) or ‘YOI’ (i.e. Young Offender Institution). Authors manually reviewed the text of all coronial files to confirm each decedent had been recently released from prison at the time of their death. The search terms were selected in collaboration with coronial staff and people with lived experience of both drug use and incarceration. Final searches of NPSUM were conducted on the 1 December 2025.

### Measures

Data on decedent socio‐demographics, mental health history (i.e. a documented history of individual mental health conditions), substance use history (i.e. documented history of substance dependence and injecting status), toxicology and which individual substances were ultimately deemed implicated in death were extracted from coronial files. All cases additionally recorded the findings concerning the single disease or condition that led directly to death, any antecedent cause(s) and other significant conditions contributory to death. Where a specific date or time of release from prison was reported, this was used to calculate the number of days that had elapsed between prison release and death. A substantial proportion of included cases did not report sufficient detail to enable calculation of this timeframe, documentation often stating only that the decedent had been ‘recently’ released from prison. For cases where the number of days were able to be calculated, decedents were categorised as having died (a) within 2 days of release (i.e. on the day of release or the following day); (b) between 2 days and 1 week following release; or (c) between 1 week and 1 month following release in line with previous classifications [1].

### Statistical analysis

We generated descriptive statistics for all decedents. Means, SDs and ranges were presented for continuous variables, as well as median and interquartile range (IQR) in skewed distributions. Overall percentages were reported for all binary and categorical variables and the valid percentage (i.e. the percentage of cases where history was available) was reported for mental health and substance use variables. We used the Joinpoint Regression Program, version 5.4.1 (US Statistical Research and Applications Branch, National Cancer Institute), to estimate the average annual percent change (AAPC) in number of deaths that occurred following prison release each year compared to the overall number of deaths reported to NPSUM in each year across the studied time period and the percentage of deaths that occurred following recent prison release in which individual substances were implicated. Weighted Bayesian information criteria (BIC) estimates used to determine best model fit. For reference, comparative data for the full NPSUM cohort can be found in the online Supporting information as Tables S2 and S3. The analysis plan was not pre‐registered and the results should be considered exploratory.

## RESULTS

A total of 597 decedents died because of a drug‐related cause following recent release from prison in the United Kingdom between 1997 and 2025. Of the 309 decedents with coronial data that enabled calculation of the timeframe of death following prison release, 140 (45.3%) were reported as dying within 2 days following release, 81 (26.2%) between 2 days and 1 week following release and 88 (28.5%) between 1 week and 1 month following release. The number of people dying because of a drug‐related cause within 1 month of release from prison per year can be seen in Figure S1. There was a statistically significant average annual percentage decrease of −3.9% (95% CI = −6.6% to −1.0%, *P* = 0.006) in the number of deaths that occurred following prison release per all deaths reported to NPSUM with the overall number of deaths observed to be highest in the 5‐year period between 2001 and 2005 (*n* = 176, 29.5%).

### Socio‐demographics and case characteristics

Socio‐demographic and case characteristics of all decedents can be found in Table 1. Overall, decedents were predominantly male (*n* = 547, 91.6%) of White ethnicity (*n* = 410, 68.7%) and had a mean age of 34.2 years (SD = 8.8; range = 16–71).

**TABLE 1:** Socio‐demographic, case characteristics, circumstances of death and mental health and substance use history of people dying because of drug‐related causes following recent release from prison in the United Kingdom, 1997–2025.

	All (*n* = 597) *n* (%)	Within 2 days of release (*n* = 140) *n* (%)	Between 2 days and 1 week following release (*n* = 81) *n* (%)	Between 1 week and 1 month following release (*n* = 88) *n* (%)
Age, mean (years)	34.2 (SD = 8.8; range = 16–71)	33.6 (SD = 7.7; range = 19–69)	33.4 (SD = 8.1; range = 19–58)	34.8 (SD = 8.7; range = 17–63)
Sex				
Female	50 (8.4)	16 (11.4)	6 (7.4)	6 (6.8)
Male	547 (91.6)	124 (88.6)	75 (92.6)	82 (93.2)
Ethnicity				
White	410 (68.7)	104 (74.3)	56 (69.1)	62 (70.5)
Black	7 (1.2)	2 (1.4)	0 (0.0)	1 (1.1)
Asian	7 (1.2)	1 (0.7)	0 (0.0)	1 (1.1)
Unknown/not recorded	173 (29.0)	33 (23.6)	25 (30.9)	24 (27.3)
Year of death^a^				
1997–2000	36 (6.0)	11 (7.9)	5 (6.2)	1 (1.1)
2001–2005	176 (29.5)	46 (32.9)	26 (32.1)	31 (35.2)
2005–2010	164 (27.5)	49 (35.0)	24 (29.6)	23 (26.1)
2011–2015	57 (9.6)	9 (6.4)	5 (6.2)	12 (13.6)
2016–2020	84 (14.1)	13 (9.3)	11 (13.6)	9 (10.2)
2021–2025	80 (13.4)	12 (8.6)	10 (12.4)	12 (13.6)
Direct cause of death (ICD‐10 code)				
Poisoning, accidental (X40–X45)	433 (72.5)	101 (72.1)	55 (67.9)	54 (61.4)
Poisoning, intentional (X60–X84)	8 (1.3)	1 (0.7)	2 (2.5)	1 (1.1)
Poisoning, undetermined intent (T40.5, Y10–Y14)	22 (3.7)	4 (2.9)	1 (1.2)	5 (5.7)
Pneumonia (J18)	16 (2.7)	1 (0.7)	3 (3.7)	2 (2.3)
Other respiratory pathology (J69, J81, J96 *etc*.)	26 (4.4)	3 (2.1)	5 (6.2)	7 (8.0)
Other (e.g. G93, W78, R99 *etc*.)	92 (15.4)	30 (21.4)	15 (18.5)	19 (21.6)
Mental health^b^				
People with a history of any mental health disorder	87 (98.9)	13 (100.0)	10 (100.0)	14 (100.0)
People with a history of a depressive disorder	45 (51.1)	4 (30.8)	7 (70.0)	10 (71.4)
Addiction^b^				
People with a history of substance dependence	518 (97.2)	118 (96.7)	70 (98.6)	79 (98.8)
People with an injecting history	138 (88.0)	33 (82.5)	22 (95.6)	26 (92.9)

Abbreviation: ICD‐10, International Classification of Diseases, 10th revision.

^a^ To August 2025.

^b^ Valid percentage reported (i.e. the percentage of cases where history was available).

### Characteristics of death and mental health and substance use history

Circumstances of death and mental health and substance use histories of all decedents can be found in Table 1. Accidental poisoning was the predominant disease or condition that was certified as the direct cause of death with coronial determination reporting 433 (72.5%) deaths directly because of accidental poisoning [International Classification of Diseases, 10th revision (ICD‐10) codes, X40–X45] and 22 (3.7%) deaths directly because of poisoning of undetermined intent (ICD‐10, Y10–Y14). Of the remaining deaths, 42 (43.2%) were reported to be directly because of pneumonia or other respiratory pathology (ICD‐10, J18, J69, J81 and J96 *etc*.) with the remaining 100 (16.8%) directly because of multiple other causes. In cases where medical history was available, the majority of decedents had a history of any mental health condition (*n* = 87/88, 98.9%), with the most common documented condition being depressive disorder (*n* = 45/88, 51.1%). The majority of decedents had a history of substance dependence (*n* = 518/533, 97.2%) with almost nine in every 10 decedents (*n* = 138/157, 88.0%) having a history of injecting.

### Number and type of drugs implicated in death

The number and type of drugs implicated in death can be found in Table 2. The median number of drugs implicated was 2 (IQR = 1–3; range = 1–7)) with multiple drug toxicity implicated in the majority of cases (*n* = 358, 60.0%). There was a statistically significant average annual percentage increase of +2.6% (95% CI = 2.4%–4.0%, *P* < 0.001) in the percentage of deaths involving multiple drug toxicity over the studied timeframe. The most commonly implicated drug group was opioids in over nine of every 10 fatalities (*n* = 545, 91.3%). There was no clear evidence of a significant AAPC (−0.003%; 95%CI = −0.03% to 0.02, *P* = 0.556) in the percentage of deaths in which opioids were implicated over the studied timeframe. The three most commonly implicated individual drugs were heroin in over three quarters of all cases (*n* = 464, 77.7%), cocaine in almost a quarter of all cases (*n* = 144, 24.1%) and alcohol in a fifth of all cases (*n* = 124, 20.8%). A third of decedents had a drug for which they had a prescription implicated in death (*n* = 207, 34.7%).

**TABLE 2:** The number and type of drugs implicated in death as deemed by the coroner among people dying because of drug‐related causes following recent release from prison in the United Kingdom, 1997–2025.

Drug implicated in death	All (*n* = 597) *n* (%)	Within 2 days of release (*n* = 140) *n* (%)	Between 2 days and 1 week following release (*n* = 81) *n* (%)	Between 1 week and 1 month following release (*n* = 88) *n* (%)
All				
Mean number of drugs implicated	2.1 (SD = 1.3; range = 1–7)	1.9 (SD = 1.1; range = 1–7)	2.1 (SD = 1.2; range = 1–6)	1.9 (SD = 1.2; range = 1–6)
Median number of drugs implicated	2 (IQR = 1–3; range =1–7)	2 (IQR = 1–2; range = 1–7)	2 (IQR = 1–3; range = 1–6)	2 (IQR = 1, 2; range = 1–6)
Only single substance implicated	239 (40.0)	56 (40.0)	31 (38.3)	40 (45.6)
Multiple substances implicated	358 (60.0)	84 (60.0)	50 (61.7)	48 (54.5)
Prescribed				
Median number of prescribed drugs implicated	0 (IQR = 0–1; range = 0–9)	0 (IQR = 0–1; range = 0–9)	0 (IQR = 0–1; range = 0–6)	0 (IQR = 0–1; range = 0–4)
Any prescribed drug implicated	207 (34.7)	42 (30.0)	24 (29.6)	28 (31.8)
Opioids				
Any opioid	545 (91.3)	130 (92.9)	77 (95.1)	78 (88.6)
Heroin	464 (77.7)	118 (84.3)	68 (83.6)	67 (76.1)
Methadone	110 (18.4)	20 (14.3)	13 (16.0)	19 (21.6)
Codeine	38 (6.4)	10 (7.1)	10 (12.3)	0 (0.0)
Dihydrocodeine	20 (3.4)	2 (1.4)	0 (0.0)	3 (3.4)
Buprenorphine	8 (1.3)	0 (0.0)	1 (1.2)	0 (0.0)
Benzodiazepines				
Any benzodiazepine	151 (25.3)	26 (18.6)	22 (27.2)	16 (18.2)
Diazepam	108 (18.1)	16 (11.4)	15 (18.5)	15 (17.0)
Gabapentinoids				
Any gabapentinoid	63 (10.6)	8 (5.7)	8 (9.9)	6 (6.8)
Antidepressants				
Any antidepressant	48 (8.0)	13 (9.3)	8 (9.9)	6 (6.8)
Cocaine	144 (24.1)	31 (22.1)	21 (25.9)	19 (21.6)
Alcohol^a^	124 (20.8)	37 (26.4)	14 (17.3)	14 (15.9)
Cannabis				
Any cannabinoid	13 (2.2)	3 (2.1)	3 (3.7)	3 (3.4)
SCRAs				
Any SCRA	9 (1.5)	3 (2.1)	0 (0.0)	0 (0.0)

Abbreviations: NPSUM, National Programme on Substance Use Mortality; IQR, interquartile range; SCRAs, synthetic cannabinoid receptor agonists.

^a^ Within NPSUM deaths in which alcohol is the only substance detected at *post‐mortem* are not recorded. As such all drug‐related deaths in which alcohol is implicated in NPSUM have, by definition, multiple substances implicated.

## DISCUSSION

Over the last three decades within the NPSUM cohort there have been a substantial number of fatalities because of drug‐related causes occurring following an individual's release from prison. Although the case characteristics are in some ways typical of UK drug‐related deaths over the same timeframe, including a high proportion of ethnically White decedents with a high percentage of mental health disorders and substance dependence, atypically over 90% of decedents were male with a mean age at death younger than 35 years old [8].

Consistent with previous studies, accidental poisoning was the most common direct cause of death, with almost three‐quarters of all deaths attributable to this preventable cause [9]. Although the evidence suggested the overall percentage of all deaths that occurred following recent prison release had decreased over the studied timeframe, the proportion of deaths in which opioids were implicated remained consistent with an increasing proportion over time implicating multiple substances. The average annual rate of imprisonment relative to general population growth has marginally increased over the studied timeframe across the United Kingdom, with an estimated 57 000 prison releases in England and Wales in 2024. [10] The observed average annual percentage decrease reported here, occurring despite this context of marginal increases in the population at risk. Potential drivers of this overall decline may include updated guidance to strengthen continuity of care between prison and community settings enacted over the study period [5]. However, although opioids were expectedly the most common class of drug implicated in death, the magnitude of their implication, in over 90% of fatalities, serves to reinforce the need to ensure the delivery of high‐quality interventions for the management of opioid use disorder (OUD) in and out of prison, including the provision of appropriately dosed opioid agonist therapy (OAT), and the provision of take‐home naloxone on release [2, 11]. These interventions have been shown to have the highest impact on mortality risk reduction within this population [11, 12].

Almost all individuals had a history of substance dependence with over four‐fifths reporting a history of injecting. Individuals deemed at any risk of return to problematic substance use following release should receive pre‐release counselling on the potential risks associated with the loss of tolerance and provided with advice on safer injecting practices [5]. The results also emphasise that individuals with a history of problematic substance use should be routinely offered linkage with community drug and alcohol treatment services on release, even if there is no current perceived prescribing need [4]. Challenges with successful and timely linkage to community treatment services, and continued OAT prescribing, have been consistently described within the literature [2, 11]. Given the substantial contribution of opioids to these deaths in the United Kingdom, substantial efforts should be made to reduce any perceived post‐release barriers to accessing treatment, improve communication between prison and community treatment services and ensure the seamless continuation of any prescribed formulation of OAT [2]. As more prison services in the United Kingdom move to the use of long‐acting injectable (LAI) depot formulations of buprenorphine as a first line treatment, this may serve to increase the time window available to attend community treatment services [13]. However, there remain concerns that individuals may not perceive as much urgency to access community treatment services if released while taking an LAI formulation. It is also important to note that, for some individuals, methadone is likely to increase retention in treatment compared to buprenorphine [14, 15]. The results also emphasise the consistent message that opioids remain the drug group overwhelmingly implicated in death at all studied time points.

The study has several strengths including the granular examination of substances implicated in death and the large national timeframe. There are, however, several important limitations of NPSUM, particularly that explanatory variables of potential interest may either not be available or may be incomplete (e.g. over a third of cases contain no reported ethnicity). A substantial number of cases did also not report sufficient detail to enable the calculation of the exact timeframe of death following release from prison with almost half of all cases only reporting that the individual had been ‘just’ released or released ‘recently’, however, reassuringly *post hoc* statistical testing revealed no significant differences in any studied outcomes between these two groups. Improved coronial documentation requiring ascertainment of the date of prison release would allow for more accurate understanding and increase statistical power. NPSUM additionally does not contain information on the individual's engagement with community treatment at the time of prison release or death. Given that treatment engagement, and the use of OAT, is protective, knowledge of the proportion of people with arranged continuity of care and accessing community services would be valuable. Future data linkage of NPSUM to treatment records may, therefore, be a fruitful avenue for further study. Because of the voluntary submission nature, NPSUM also has incomplete coverage of the UK coronial system. Although over 90% of coroners’ report, non‐reporting jurisdictions contain heavily populated areas with previous triangulation work estimating that NPSUM receives between 65% and 70% of all drug poisoning deaths officially reported by the UK Government Office of National Statistics (ONS) [16]. There is additional variability of toxicological examination across cases, therefore, some deaths will likely have been missed and some implicated substances not identified [7]. Although the overall numbers of deaths following prison release should, therefore, be taken as conservative estimates, the strength of NPSUM remains as the only data source currently able to granularly identify individual substances implicated in death.

In conclusion, there have been a substantial number of deaths because of drug‐related causes among individuals recently released from prison over the past three decades in the United Kingdom. Opioids were the drug group implicated in the highest proportion of deaths, involved in over 90% of fatalities. Facilitating linkage to community treatment services and ensuring access to timely, evidence‐based interventions for the diagnosis and management of opioid use disorder should be a priority. Professionals and people who use drugs should be aware of the substantial elevated risk of drug‐related death following release from prison and the minimal observed differences in the characteristics of death occurring throughout the month following release.

## AUTHOR CONTRIBUTIONS

**Emmert Roberts:** Conceptualization; investigation; funding acquisition; writing—original draft; methodology; formal analysis; project administration; resources. **John Strang:** Investigation; writing—review and editing; validation; supervision. **Caroline Copeland:** Investigation; writing—review and editing; validation; project administration; data curation; supervision; resources.

## DECLARATION OF INTERESTS

All authors have completed the ICJME Unified Competing Interest form (available on request from the corresponding author) and declare: no support from any organisation for the submitted work; no financial relationships with organisations that might have an interest in the submitted work in the previous 3 years; and no other relationships or activities that could appear to have influenced the submitted work.

## Supporting information


**Figure S1.** The number of people dying per year due to drug‐related causes within one month of release from prison in the United Kingdom, 1997–2025.
**Table S1.** Strengthening the Reporting of Observational Studies in Epidemiology (STROBE) statement checklist [1].
**Table S2.** Percentage of sociodemographic, case characteristics, circumstances of death and mental health and substance use history of people dying due to drug‐related causes in the National Programme on Substance Use Mortality (NPSUM) in the United Kingdom, 1997–2025.
**Table S3.** Percentage and type of drugs implicated in death among people dying due to drug‐related causes in the National Programme on Substance Use Mortality (NPSUM) in the United Kingdom, 1997–2025.

## Data Availability

The data that support the findings of this study are available on request from the corresponding author. The data are not publicly available due to privacy or ethical restrictions.

## References

[1] Merrall EL , Kariminia A , Binswanger IA , Hobbs MS , Farrell M , Marsden J , et al. Meta‐analysis of drug‐related deaths soon after release from prison. Addiction. 2010;105(9):1545–1554. 10.1111/j.1360-0443.2010.02990.x 20579009 PMC2955973

[2] Marsden J , Stillwell G , Jones H , Cooper A , Eastwood B , Farrell M , et al. Does exposure to opioid substitution treatment in prison reduce the risk of death after release? A national prospective observational study in England. Addiction. 2017;112(8):1408–1418. 10.1111/add.13779 28160345

[3] Binswanger IA , Blatchford PJ , Mueller SR , Stern MF . Mortality after prison release: opioid overdose and other causes of death, risk factors, and time trends from 1999 to 2009. Annals of Internal Medicine. 2013;159(9):592–600. 10.7326/0003-4819-159-9-201311050-00005 24189594 PMC5242316

[4] Binswanger IA , Stern MF , Deyo RA , Heagerty PJ , Cheadle A , Elmore JG , et al. Release from prison—a high risk of death for former inmates. New England Journal of Medicine. 2007;356(2):157–165. 10.1056/NEJMsa064115 17215533 PMC2836121

[5] Lewer D , Edge C . Preventing deaths after prison release. The Lancet. 2024;403(10438):1727–1729. 10.1016/S0140-6736(24)00652-4 38614114

[6] Von Elm E , Altman DG , Egger M , Pocock SJ , Gøtzsche PC , Vandenbroucke JP . The strengthening the reporting of observational studies in epidemiology (STROBE) statement: Guidelines for reporting observational studies. Annals of Internal Medicine. 2007;147(8):573–577. 10.7326/0003-4819-147-8-200710160-00010 17938396

[7] Roberts E , Copeland C , Humphreys K , Shover CL . Drug‐related deaths among housed and homeless individuals in the UK and the USA: Comparative retrospective cohort study. The British Journal of Psychiatry. 2023;223(6):1–7. 10.1192/bjp.2023.111 PMC10727910 37665046

[8] https://www.ons.gov.uk/peoplepopulationandcommunity/birthsdeathsandmarriages/deaths/bulletins/deathsrelatedtodrugpoisoninginenglandandwales/previousReleases

[9] Bukten A , Stavseth MR , Skurtveit S , Tverdal A , Strang J , Clausen T . High risk of overdose death following release from prison: variations in mortality during a 15‐year observation period. Addiction. 2017;112(8):1432–1439. 10.1111/add.13803 28319291

[10] Prison population statistics. https://commonslibrary.parliament.uk/research-briefings/sn04334/

[11] Strang J , Bird SM , Parmar MK . Take‐home emergency naloxone to prevent heroin overdose deaths after prison release: Rationale and practicalities for the N‐ALIVE randomized trial. Journal of Urban Health. 2013;90(5):983–996. 10.1007/s11524-013-9803-1 23633090 PMC3795186

[12] Hedrich D , Alves P , Farrell M , Stöver H , Møller L , Mayet S . The effectiveness of opioid maintenance treatment in prison settings: a systematic review. Addiction. 2012;107(3):501–517. 10.1111/j.1360-0443.2011.03676.x 21955033

[13] Sayers C , Mogford D . Patient satisfaction and resource utilization following introduction of long‐acting injectable buprenorphine (LAIB) in Scottish prisons. Substance Abuse and Rehabilitation. 2025;16:83–93. 10.2147/SAR.S510467 40256212 PMC12009042

[14] Degenhardt L , Clark B , Macpherson G , Leppan O , Nielsen S , Zahra E , et al. Buprenorphine versus methadone for the treatment of opioid dependence: A systematic review and meta‐analysis of randomised and observational studies. Lancet Psychiatry. 2023;10(6):386–402. 10.1016/S2215-0366(23)00095-0 37167985

[15] Roselló‐Jordá S , Espinós‐Navarro C , Molés‐Julio MP . Effectiveness of opioid maintenance programs for treating drug dependence in prisons. Revista Espanola de Sanidad Penitenciaria. 2023;25(3):112–121. 10.18176/resp.00077 38289166 PMC10910325

[16] Chen S , Mais D , Copeland CS . Comparison of Office for National Statistics (ONS) and National Programme on substance use mortality (NPSUM) data suggests that opioid‐related deaths in England & Wales have been systematically underestimated. The International Journal on Drug Policy. 2025;145:104976. 10.1016/j.drugpo.2025.104976 40886523

