# Supplementary material for: Characteristics of deaths due to drug‐related causes among individuals recently released from prison in the United Kingdom, 1997–2025
Source: Addiction. 2026 May 14;121(9):2545–51. doi: 10.1111/add.70481 (PMC13486934; doi:10.1111/add.70481)
Supplement: Supplementary file 1 [file ADD-121-2545-s001.docx]

**Online Supplementary Material**

Contents

Figures

S1. The number of people dying per year due to drug-related causes within one month of release from prison in the United Kingdom, 1997 – 2025

Tables

S1. Strengthening the Reporting of Observational Studies in Epidemiology (STROBE) statement checklist (1)

S2. Percentage of sociodemographic, case characteristics, circumstances of death and mental health and substance use history of people dying due to drug-related causes in the National Programme on Substance Use Mortality (NPSUM) in the United Kingdom, 1997 - 2025

S3. Percentage and type of drugs implicated in death among people dying due to drug-related causes in the National Programme on Substance Use Mortality (NPSUM) in the United Kingdom, 1997 - 2025

References

Figures

Figure S1: The number of people dying per year due to drug-related causes within one month of release from prison in the United Kingdom, 1997 – 2025

a) The raw number of people dying per year due to drug-related causes within one month of release from prison in the United Kingdom, 1997 – 2025

Year (*until August 2025)

Raw number of people dying due to drug-related causes within one month of release from prison in the United Kingdom

b) The number of people dying per year due to drug-related causes within one month of release from prison per the overall number of people dying per year due to drug drug-related causes reported to the National Programme on Substance Use Mortality (NPSUM) in the United Kingdom, 1997 – 2025

Year (*until August 2025)

Number of people dying due to drug-related causes within one month of release from prison per the overall number of people dying due to drug related causes reported to the NPSUM in the United Kingdom

Average Annual Percentage Change (AAPC)

-3.9% (95%CI -6.6% to -1.0%, p=0.006)

c) The percentage of deaths per year due to drug-related causes that occurred within one month of release from prison in which opioids were implicated in the United Kingdom, 1997 – 2025

Year (*until August 2025)

Average Annual Percentage Change (AAPC)

-0.003%, 95%CI -0.03% to +0.02, p=0.556)

Percentage of deaths due to drug-related causes within one month of release from prison in which opioids were implicated in the United Kingdom

d) The percentage of deaths per year due to drug-related causes that occurred within one month of release from prison in which multiple substances (i.e., > 1) were implicated in the United Kingdom, 1997 – 2025

Year (*until August 2025)

Percentage of deaths due to drug-related causes within one month of release from prison in which multiple substances were implicated in the United Kingdom

Average Annual Percentage Change (AAPC)

+2.6% (95%CI +2.4% to +4.0%, p<0.001)

Tables

Table S1: Strengthening the Reporting of Observational Studies in Epidemiology (STROBE) statement checklist (1)

|  | Item No | Recommendation |
| --- | --- | --- |
| **Title and abstract** | 1 | (*a*) Indicate the study’s design with a commonly used term in the title or the abstract; Title: Page 1 |
|  |  | (*b*) Provide in the abstract an informative and balanced summary of what was done and what was found; Abstract: Page 3 |
| Introduction | | |
| Background/rationale | 2 | Explain the scientific background and rationale for the investigation being reported; Introduction: Page 5 |
| Objectives | 3 | State specific objectives, including any prespecified hypotheses; Introduction: Page 5 |
| Methods | | |
| Study design | 4 | Present key elements of study design early in the paper; Methods: Page 6 |
| Setting | 5 | Describe the setting, locations, and relevant dates, including periods of recruitment, exposure, follow-up, and data collection; Methods: Pages 6/7 |
| Participants | 6 | (*a*) Give the eligibility criteria, and the sources and methods of selection of participants. Describe methods of follow-up; Methods: Pages 6/7 |
|  |  | (*b*) For matched studies, give matching criteria and number of exposed and unexposed N/A |
| Variables | 7 | Clearly define all outcomes, exposures, predictors, potential confounders, and effect modifiers. Give diagnostic criteria, if applicable Methods: Page 6/7 |
| Data sources/ measurement | 8* | For each variable of interest, give sources of data and details of methods of assessment (measurement). Describe comparability of assessment methods if there is more than one group Methods: Page 6/7 |
| Bias | 9 | Describe any efforts to address potential sources of bias Methods: Page 6 |
| Study size | 10 | Explain how the study size was arrived at Methods Page 6/7 ; Results Page 9 |
| Quantitative variables | 11 | Explain how quantitative variables were handled in the analyses. If applicable, describe which groupings were chosen and why Methods Page 7/8 |
| Statistical methods | 12 | (*a*) Describe all statistical methods, including those used to control for confounding Methods Page 7/8 |
|  |  | (*b*) Describe any methods used to examine subgroups and interactions N/A |
|  |  | (*c*) Explain how missing data were addressed Methods Page 7/8 |
|  |  | (*d*) If applicable, explain how loss to follow-up was addressed N/A |
|  |  | (*e*) Describe any sensitivity analyses N/A |
| Results | | |
| Participants | 13* | (a) Report numbers of individuals at each stage of study—eg numbers potentially eligible, examined for eligibility, confirmed eligible, included in the study, completing follow-up, and analysed Results Page 9 |
|  |  | (b) Give reasons for non-participation at each stage N/A |
|  |  | (c) Consider use of a flow diagram N/A |
| Descriptive data | 14* | (a) Give characteristics of study participants (eg demographic, clinical, social) and information on exposures and potential confounders Results Pages 9; Table One |
|  |  | (b) Indicate number of participants with missing data for each variable of interest Table One |
|  |  | (c) Summarise follow-up time (eg, average and total amount) Methods Page 6/7 |
| Outcome data | 15* | Report numbers of outcome events or summary measures over time Table one/Table two |
| Main results | 16 | (*a*) Give unadjusted estimates and, if applicable, confounder-adjusted estimates and their precision (eg, 95% confidence interval). Make clear which confounders were adjusted for and why they were included Table one/ Table two |
|  |  | (*b*) Report category boundaries when continuous variables were categorized Table two |
|  |  | (*c*) If relevant, consider translating estimates of relative risk into absolute risk for a meaningful time period N/A |
| Other analyses | 17 | Report other analyses done—eg analyses of subgroups and interactions, and sensitivity analyses Table 1 Results Pages 9/10 |
| Discussion | | |
| Key results | 18 | Summarise key results with reference to study objectives Discussion Page 11 |
| Limitations | 19 | Discuss limitations of the study, taking into account sources of potential bias or imprecision. Discuss both direction and magnitude of any potential bias Discussion Page 12 |
| Interpretation | 20 | Give a cautious overall interpretation of results considering objectives, limitations, multiplicity of analyses, results from similar studies, and other relevant evidence Discussion Page 13 |
| Generalisability | 21 | Discuss the generalisability (external validity) of the study results Discussion Page 11/12 |
| Other information | | |
| Funding | 22 | Give the source of funding and the role of the funders for the present study and, if applicable, for the original study on which the present article is based Title Page 2 |

Table S2: Percentage of sociodemographic, case characteristics, circumstances of death and mental health and substance use history of people dying due to drug-related causes in the National Programme on Substance Use Mortality (NPSUM) in the United Kingdom, 1997 - 2025

|  | | All n=58,377  % |  | | All n=58,377  % |
| --- | --- | --- | --- | --- | --- |
| **Age** | Mean (Years) | 41.0 (SD 13.4; Range 0 - 105) | **Year of Death** | 1995 - 2000 | 8.6 |
| **Sex** | Female | 26.8 |  | 2001 - 2005 | 16.0 |
|  | Male | 73.2 |  | 2005 - 2010 | 19.0 |
| **Ethnicity** | White | 64.5 |  | 2011 - 2015 | 15.9 |
|  | Black | 0.9 |  | 2016 - 2020 | 19.8 |
|  | Asian | 0.7 |  | 2021 - 2025 | 20.8 |
|  | Unknown/Not Recorded | 33.9 | **Addiction^2^** | People with a history of substance dependence | 76.8 |
| **Occupation^1^** | Employed | 24.3 |  | People with an injecting history | 53.8 |
|  | Unemployed | 43.2 |  |  |  |
|  | Childcare/Houseperson | 1.2 |  |  |  |
|  | Student/Pupil | 1.7 |  |  |  |
|  | Retired/Invalid/Sickness | 9.2 |  |  |  |
|  | Unknown | 20.3 |  |  |  |

^SD Standard Deviation; 1 Occupation definitions are based on those used by the United Kingdom Office of National Statistics (ONS) (2); 2 Valid percentage reported^

Table S3: Percentage and type of drugs implicated in death among people dying due to drug-related causes in the National Programme on Substance Use Mortality (NPSUM) in the United Kingdom, 1997 - 2025

| Drug implicated in death | | All n=58,377  % |
| --- | --- | --- |
| All | Mean number of drugs implicated | 2.1 (SD 1.5; Range 0 - 14) |
|  | Median number of drugs implicated | 2 |
|  | Only single substance implicated | 38.2 |
|  | Multiple substances implicated | 61.8 |
| Opioids | Any opioid | 66.9 |
|  | Heroin | 41.1 |
|  | Methadone | 19.1 |
|  | Dihydrocodeine | 4.7 |
| Benzodiazepines | Any benzodiazepine | 19.4 |
|  | Diazepam | 14.7 |
| Antidepressants | Any antidepressant | 17.3 |
| Cocaine | Cocaine | 16.9 |
| Alcohol^1^ | Alcohol | 24.6 |
| Synthetic Cannabinoid Receptor Agonists (SCRAs) | Any SCRA | 0.7 |

^SD Standard Deviation; IQR Inter-quartile range;1 Within NPSUM deaths in which alcohol is the only substance detected at post-mortem^

^are not recorded.^

**References**

1.	Von Elm E, Altman DG, Egger M, Pocock SJ, Gøtzsche PC, Vandenbroucke JP. The Strengthening the Reporting of Observational Studies in Epidemiology (STROBE) statement: guidelines for reporting observational studies. Annals of internal medicine. 2007;147(8):573-7.

2.	<https://www.ons.gov.uk/methodology/classificationsandstandards/otherclassifications/thenationalstatisticssocioeconomicclassificationnssecrebasedonsoc2010>.
